# miR-1 and miR-133b Are Differentially Expressed in Patients with Recurrent Prostate Cancer

**DOI:** 10.1371/journal.pone.0098675

**Published:** 2014-06-26

**Authors:** Omer Faruk Karatas, Esra Guzel, Ilknur Suer, Isin D. Ekici, Turhan Caskurlu, Chad J. Creighton, Michael Ittmann, Mustafa Ozen

**Affiliations:** 1 Department of Medical Genetics, Istanbul University, Cerrahpasa Medical School, Istanbul, Turkey; 2 Molecular Biology and Genetics Department, Erzurum Technical University, Erzurum, Turkey; 3 Biruni University, Istanbul, Turkey; 4 Department of Medical Pathology, Yeditepe University, Istanbul, Turkey; 5 Departmentof Urology, Goztepe Education and Research Hospital, Goztepe, Istanbul, Turkey; 6 Division of Biostatistics, Baylor College of Medicine, Houston, Texas, United States of America; 7 Department of Pathology & Immunology, Baylor College of Medicine, and Michael E. DeBakey VAMC, Houston, Texas, United States of America; The University of Hong Kong, China

## Abstract

Prostate cancer (PCa) is currently the most frequently diagnosed malignancy in the western countries. It is more prevalent in older men with 75% of the incident cases above 65 years old. After radical prostatectomy, approximately 30% of men develop clinical recurrence with elevated serum prostate-specific antigen levels. Therefore, it is important to unravel the molecular mechanisms underlying PCa progression to develop novel diagnostic/therapeutic approaches. In this study, it is aimed to compare the microRNA (miRNA) profile of recurrent and non-recurrent prostate tumor tissues to explore the possible involvement of miRNAs in PCa progression. Total RNA from 41 recurrent and 41 non-recurrent PCa tissue samples were used to investigate the miRNA signature in PCa specimens. First of all, 20 recurrent and 20 non-recurrent PCa samples were profiled using miRNA microarray chips. Of the differentially expressed miRNAs, miR-1, miR-133b and miR-145* were selected for further validation with qRT-PCR in a different set of 21 recurrent and 21 non-recurrent PCa samples. Data were statistically analyzed using two-sided Student's t-test, Pearson Correlation test, Receiver operating characteristic analysis. Our results demonstrated that miR-1 and mir-133b have been significantly downregulated in recurrent PCa specimens in comparison to non-recurrent PCa samples and have sufficient power to distinguish recurrent specimens from non-recurrent ones on their own. Here, we report that the relative expression of miR-1 and mir-133b have been significantly reduced in recurrent PCa specimens in comparison to non-recurrent PCa samples, which can serve as novel biomarkers for prediction of PCa progression.

## Introduction

Prostate cancer (PCa) is currently the most frequently diagnosed malignancy and the second leading cause of cancer deaths among men over the age of 50 years in the western countries [1]. PCa has a propensity to be much more prevalent in older men with 75% of the incident cases above 65 years old [2]. Radical prostatectomy, radical radiotherapy and hormone ablation therapy are the preferentially applied techniques for early-stage clinically localized tumors to cure the disease, however, these techniques do not necessarily provide enhanced survival rates [3] and almost 30% of men develop clinical recurrence with increased serum prostate-specific antigen (PSA) levels [4]. On the other hand, for advanced and metastatic tumors, chemotherapy, which is the only option, mostly fails to give positive clinical outcome [5]. Therefore it is of great importance to understand the molecular mechanisms underlying prostate cancer progression to develop novel diagnostic and therapeutic approaches.

MicroRNAs (miRNA) are endogenously synthesized, regulatory non-coding small RNAs comprised of around 20 nucleotides and considered as a novel class of gene regulators. They suppress expression of their targets through mRNA degradation or translational inhibition as a result of incomplete binding to the 3′untranslated regions (3′UTR) of mRNAs [6]. MiRNAs are supposed to regulate the expression of almost 60% of human genes [7]. About half of the annotated human miRNAs are located in fragile sites of the genome suggesting that these small molecules might have a vital function in pathogenesis of several diseases including cancer [8], [9]. They have been shown to be able to distinguish differentiation states of several malignancies including breast, lung and colon cancers. In addition, there are studies suggesting miRNA expression profiling can distinguish malignant from non-malignant prostate tissues [10], [11]. There are, however, a limited number of reports in the literature studied miRNAs in prostate cancer progression.

MiRNA profiling through microarrays is an invaluable technique to determine a miRNA signature, which is necessarily significant to figure out the general and specific expression alterations between distinct types of tissues [12], [13]. In this study, we aimed to compare the miRNA profile of recurrent and non-recurrent prostate tumor tissues for shedding light upon a possible involvement of miRNAs in PCa progression.

## Materials and Methods

### Patients

RNAs were isolated from 41 recurrent and 41 non-recurrent cancers from radical prostatectomies, which contained at least 70% tumor tissue, which were obtained from Baylor College of Medicine Prostate Cancer program. Recurrence was defined as two consecutive serum PSAs greater than 0.2 ng/ml. Patients were followed for until PSA recurrence or at least 4 years (for non-recurrent cases). This study has been approved by an internal institutional review board of Baylor College of Medicine. Patients were included into study upon giving their written informed consent. The characteristics of the recurrent and non-recurrent patients are summarized in Table 1.

**Table 1:** Race/ethnicity, age, Gleason Score, PSA values, SVI (Seminal Vesicle Invasion) of recurrent and non-recurrent PCa patients that are involved in the study.

LAB ID	Race/Ethnicity	AGE	Gleason Score	PSA	SVI
**R1**	Caucasian	71.9	3+4	24.7	0
**R2**	Hispanic	64.1	4+4	9.1	1
**R3**	Caucasian	63.8	3+4	5.6	1
**R4**	Caucasian	58	4+3	3.6	1
**R5**	Caucasian	66.5	4+4	-	1
**R6**	Caucasian	66.2	3+4	-	0
**R7**	Caucasian	61.3	3+4	16.8	0
**R8**	-	66	4+3	-	0
**R9**	Caucasian	69.6	4+4	-	0
**R10**	Caucasian	58.8	4+4	41	0
**R11**	Caucasian	60	4+4	-	1
**R12**	African American	63.5	4+4	100	-
**R13**	Caucasian	57.9	3+4	77.6	1
**R14**	Caucasian	42.7	3+4	31	1
**R15**	Caucasian	65.8	2+3	-	0
**R16**	Caucasian	58.2	3+4	-	1
**R17**	Caucasian	60.9	3+4	9.8	0
**R18**	Caucasian	59.9	3+4	8.1	1
**R19**	Caucasian	60.6	3+4	26	0
**R20**	Caucasian	54.1	4+3	24.7	1
**R21**	Caucasian	63.8	4+3	17.3	1
**R22**	Caucasian	60.6	3+4	26	0
**R23**	Caucasian	59.9	3+4	8.1	1
**R24**	-	63	4+3	-	1
**R25**	Caucasian	64.6	3+4	-	1
**R26**	Caucasian	64.7	3+4	5.3	0
**R27**	Caucasian	59.5	3+4	5.1	0
**R28**	Caucasian	70.8	2+4	-	0
**R29**	Caucasian	56.9	3+3	48	0
**R30**	Caucasian	57.6	3+4	4.3	0
**R31**	Caucasian	69.3	4+3	32	0
**R32**	Caucasian	72.6	3+4	4.2	0
**R33**	Caucasian	72.2	3+4	17.1	0
**R34**	Caucasian	65.1	4+4	-	0
**R35**	Caucasian	75.7	3+4	10	0
**R36**	Caucasian	59.3	3+3	5.7	0
**R37**	Caucasian	66.6	4+3	-	0
**R38**	Caucasian	58.6	4+5	34.8	1
**R39**	Caucasian	60.9	2+4	8.6	0
**R40**	African American	56.2	4+4	12.8	0
**R41**	Caucasian	66.2	3+4	-	0
**NR1**	Caucasian	68	3+3	3.1	0
**NR2**	Caucasian	54.5	3+4	15.6	0
**NR3**	Caucasian	69.7	3+4	10.4	0
**NR4**	Caucasian	66.7	3+4	11.1	0
**NR5**	Hispanic	58.8	3+4	8.2	0
**NR6**	Caucasian	59.5	3+3	-	0
**NR7**	-	53	3+3	-	0
**NR8**	Caucasian	63.3	3+4	-	0
**NR9**	Caucasian	65.5	4+3	8.2	0
**NR10**	Hispanic	59	3+3	-	0
**NR11**	-	58	3+4	-	0
**NR12**	-	59	4+3	-	0
**NR13**	Hispanic	64.1	3+4	9	0
**NR14**	Caucasian	73.8	3+3	3.4	0
**NR15**	Caucasian	67.2	3+4	9.5	0
**NR16**	Hispanic or Latino	62.1	4+3	32.6	0
**NR17**	Caucasian	67.6	4+3	6.6	1
**NR18**	Caucasian	72.5	4+3	3.2	0
**NR19**	Caucasian	55.7	3+3	-	0
**NR20**	-	53	3+3	-	0
**NR21**	Caucasian	60.2	4+3	13.6	0
**NR22**	Caucasian	56.3	3+4	40.3	1
**NR23**	Caucasian	67.1	3+3	6.6	0
**NR24**	Caucasian	63.6	4+3	-	0
**NR25**	Caucasian	60.1	3+4	8	0
**NR26**	Caucasian	54.4	2+3	-	1
**NR27**	Caucasian	70.1	3+4	8.2	0
**NR28**	Caucasian	67.2	3+4	9.5	0
**NR29**	Caucasian	67.1	3+4	4.4	0
**NR30**	Caucasian	40.1	4+4	7.2	0
**NR31**	Caucasian	54.6	3+3	-	0
**NR32**	Caucasian	59.3	3+3	-	0
**NR33**	Caucasian	55.7	3+3	-	0
**NR34**	Caucasian	71	5+5	-	1
**NR35**	Caucasian	50.8	3+3	9	0
**NR36**	Caucasian	57.8	3+3	4.7	0
**NR37**	African American	52	3+3	5	0
**NR38**	Caucasian	65.4	3+4	-	0
**NR39**	Caucasian	72	3+4	-	0
**NR40**	Caucasian	70.2	3+3	-	0
**NR41**	Hispanic	62.2	4+3	32.6	0

“-” represents “not available”. For SVI, “0” represents “no invasion” and “1” represents “invasion”.

### Total RNA Isolation

Total RNA from 41 recurrent and 41 non-recurrent tissue samples using Trizol (Invitrogen, San Diego, CA) reagent according to the manufacturer's instructions. The purities and concentrations of RNA samples were determined spectrophotometrically using NanoDrop ND-2000c (Thermo Fisher Scientific, Inc., Wilmington, DE). RNA integrity was tested using gel electrophoresis and Spot Check Nucleic Acid Quantitation Kit (Sigma).

### MiRNA Microarray and Data Analysis

100 ng of total RNA from 20 recurrent and 20 non-recurrent samples are labeled with Cy3 by using Agilent miRNA labeling kit following manufacturer's protocol. Labeled RNAs are heat denatured and hybridized to Agilent 8×15 k miRNA microarray V2 comprised of 799 probes targeting a comprehensive selection of 723 human and 76 human viral miRNAs with control probes from Sanger miRBase (release 10.1) at 55°C for 20 hr. After hybridization and post-hybridization washes, slides were scanned immediately in Agilent Microarray Scanner with Surescan High Resolution Technology (Agilent Technologies, Santa Clara, CA). Feature Extraction v10.7.3.1 (Agilent Technologies, CA) software was used to extract all features of the data obtained from the scanned images and Bioconductor software was used to analyze the raw data, which were normalized by quantile normalization. P values (by two-sided t-test) and fold changes between comparison groups were calculated, using log-transformed data. Out of 15714 probes, the number of true positives would need to greatly exceed 157 for the expected False Discovery Rate (FDR) to be considered low (using the method by Storey et al. [14]). The nominally significant probes did not exceed chance expected by multiple testing, which necessitated the additional validation of select miRNAs. Array data have been deposited into the Gene Expression Omnibus (GEO, accession number GSE55323).

### cDNA Synthesis and Quantitative Real-Time PCR

To validate the differential expression of miR-1, miR-133b and miR-145*, RNA samples from a different set of 21 recurrent and 21 non-recurrent patients were studied. For miRNA qRT-PCR experiments, equal amounts of total RNA (30 ng) from each sample was used for first strand DNA (cDNA) synthesis using miRNA specific primers purchased from Applied Biosystems and “TaqMan MicroRNA reverse transcription Kit” according to the manufacturer's protocol (Applied Biosystems, Foster City, CA). TaqMan hsa-miR-1 (Assay ID: 002222), −133b (Assay ID: 002247) and −145* (Assay ID: 002149) amplification kits were obtained from Applied Biosystems (Foster City, CA).

MiRNA expression analysis by quantitative RT-PCR was carried out using a Roche LightCycler480-II real-time thermal cycler (Roche, Switzerland). TaqMan Universal Master Mix (Applied Biosystems, Foster City, CA) was used and microRNA specific probes were purchased from Applied Biosystems (Denmark). MiRNA expression data were normalized to RNU43. Each experiment was performed in duplicate. The relative quantification analysis was performed by delta-delta-Ct method as described previously [15].

### Statistical Analysis

Statistical analysis was performed using two-sided Student's t-test. A p-value<0.05 was considered as statistically significant. Pearson Correlation test was used to show the correlation of miR-1 and miR-133b expression in PCa specimens. Receiver operating characteristic (ROC) curves were plotted using SPSS 15.0 to see the power of PSA and validated miRNAs to differentiate the recurrent PCa samples from non-recurrent samples. For ROC analysis, the logistic regression was conducted and the predicted probabilities were calculated for each single miRNA or miR-1 and miR-133b together. Then, the area under the curve is calculated with 95% confidence interval. The area under the curve is accepted significantly different from 0.5 when p-value is greater than 0.5 meaning that the logistic regression classifies the group significantly better than by chance.

## Results

A total of 41 recurrent and 41 non-recurrent tumors from radical prostatectomies, which were obtained from Baylor College of Medicine Prostate Cancer program, were included in this study to perform miRNA profiling (Table 1). The average age at the time of surgery of the PCa patients with recurrence were 62.8±6, whereas those without recurrence had an average age of 61.7±7.2 (not significant, p = .54, Mann Whitney). More than 80% of patients in both groups were non-Hispanic Caucasians. Average months from operation to 1^st^ recurrence or last normal evaluation for recurrent and non-recurrent PCa patients were 22.63±3.89 and 76.59±2.87, respectively. PSA levels ranges from 4.2 to 100 and 3.1 to 40.3 in patients with recurrence and non-recurrence, respectively. As expected, mean pre-operative PSA level of recurrent patients was almost twice that of non-recurrent patients (22 vs. 11.3 ng/ml). In recurrent patients only 3 samples were Gleason 6 (3 of 41) while for non-recurrent patients 14 of 41 samples were Gleason 5 or 6. Similarly, 15 of 40 radical prostatectomies from recurrent patients for which data was available showed seminal vesicle invasion but only 4 of 41 non-recurrent cancers had this feature. Thus while the demographic features of the two groups are very similar, the recurrent cancers are much more highly aggressive based on clinical and pathological features.

To compare the miRNA profiles of recurrent and non-recurrent PCa specimens, we carried out microarray analysis using 20 samples from each group. Microarray analysis of 20 recurrent and 20 non-recurrent samples revealed that 93 probes have been differentially expressed with a p value less than 0.01. Of the 93 nominally significant probes reported, there are 84 mapping to human miRNAs, which are provided in Table S1.

**Figure 1:**
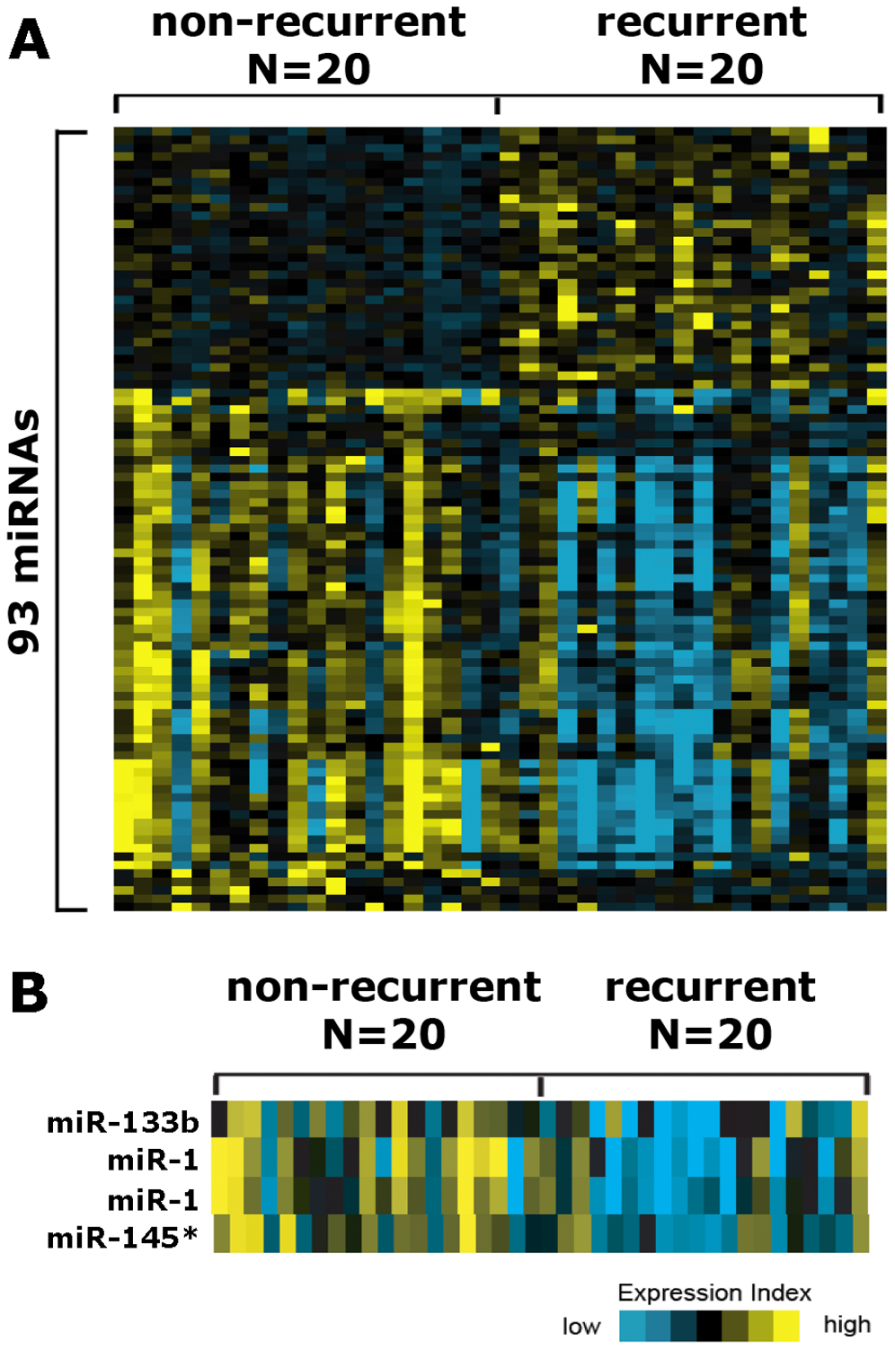
Heatmap representation of significantly deregulated miRNAs. (A) Heat-map representation of significantly deregulated miRNAs in recurrent PCa specimens vs. non-recurrent PCa specimens. (B) Heat-map representation of miR-1, miR-133b and miR-145* in recurrent PCa specimens vs. non-recurrent PCa specimens.

A heat map representation of differentially expressed miRNAs is demonstrated in Figure 1A. Among the significantly deregulated miRNAs, mir-1, mir-133b, and mir-145* (Figure 1B) were selected for further qRT-PCR confirmation in a different set of recurrent and non-recurrent PCa samples.

**Figure 2:**
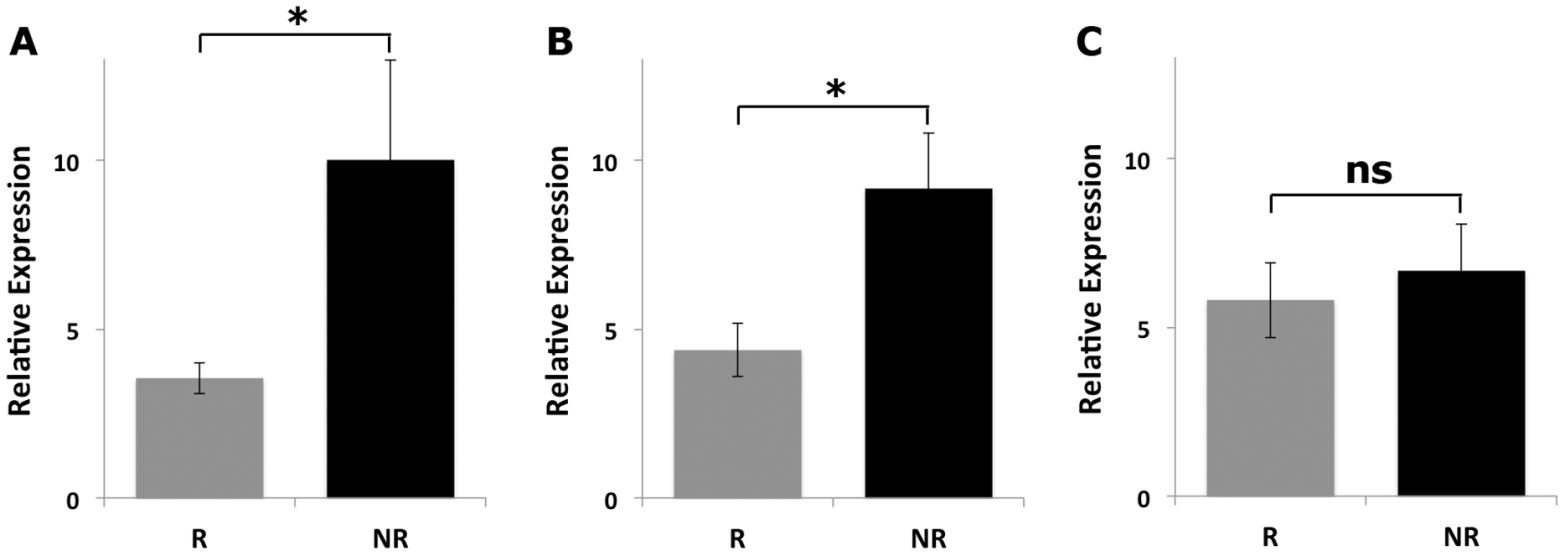
Relative expression levels of miR-1, miR-133b, and miR-145*. Relative expression levels of (A) miR-1, (B) miR-133b, and (C) miR-145* in 20 recurrent PCa specimens compared to 20 non-recurrent PCa specimens. RNU43 was used for normalization of miRNA expression analyses.

**Figure 3:**
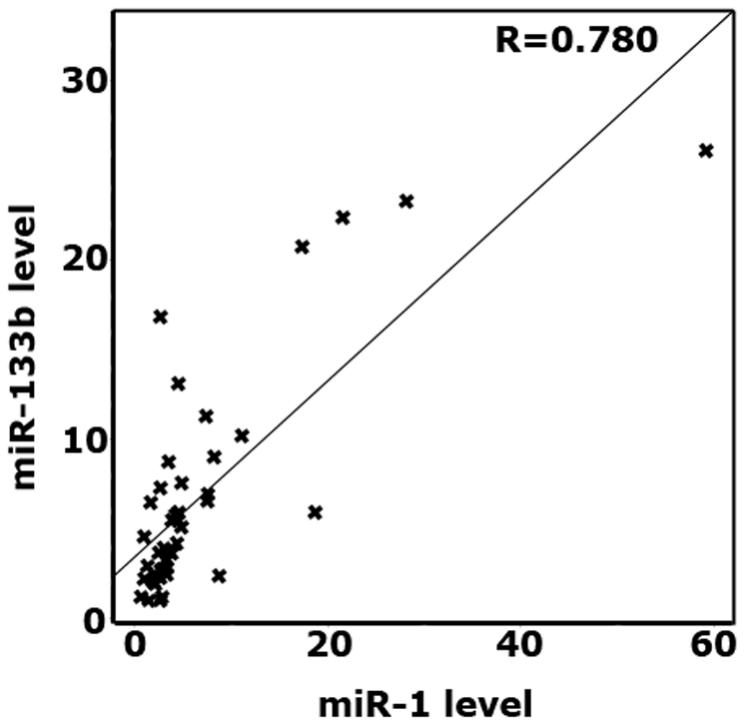
Pearson Correlation analysis of miR-1 and miR-133b.

The qRT-PCR results demonstrated that the expression level of miR-1 was significantly reduced in recurrent PCa specimens than that of non-recurrent PCa tissue samples (Figure 2A, p = 0.036). Downregulation of mir-133b in recurrent PCa (Figure 2B, p = 0.012) was also confirmed. On the contrary, although there is a slight decrease in the expression level of miR-145* in recurrent specimens as compared to non-recurrent ones, the difference was not statistically significant (Figure 2C, p>0.05). To evaluate the correlation of miR-1 and miR-133b expression in both recurrent and non-recurrent PCa specimens, we utilized Pearson correlation analysis, which demonstrated that miR-1 expression was strongly correlated with miR-133b expression in PCa tissue samples (Figure 3, correlation coefficient (R) = 0.780).

**Figure 4:**
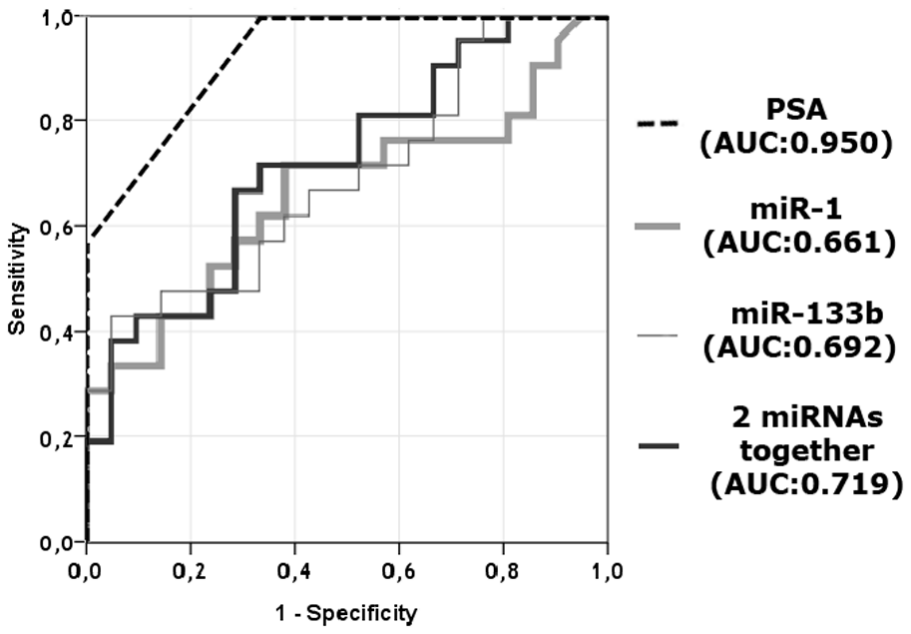
ROC analysis of miR-1 and miR-133b. Curves for individual miRNAs and their cooperative power to discriminate two sets of patients comprised of 20 recurrent and 20 non-recurrent PCa specimens.

To test the power of the PSA, miR-1 and miR-133b for distinguishing recurrent PCa specimens from non-recurrent samples, receiver operating curves (ROC) were plotted, which showed that PSA, miR-1 and miR-133b had area under the curve (AUC) values of 0.950, 0.661 and 0.692, respectively, which demonstrates their sufficiency to have the power to distinguish recurrent specimens from non-recurrent ones on their own. Moreover, when miR-1 and miR-133b were evaluated together they have represented better power (AUC; 0.719) than the case where miRNAs were analyzed individually (Figure 4).

## Discussion

PCa is a highly heterogeneous disease, for which current prognostic indicators mostly fail to determine the outcome and almost 30% of the PCa patients experience a relapse after a successful radical prostatectomy or adjuvant therapy [16], [17]. Currently, primary tumor stage, serum PSA level, and biopsy Gleason grade are utilized during clinical assessment to predict the pathologic stage of the tumor and the treatment efficiency, however, none or even a combination of these indicators are sufficient to reliably anticipate outcome for patients [3], [4]. A consensus criteria on the definition of biochemical recurrence based on PSA levels, has not been well established, which prevents the establishment of a standard prognostic model in men treated with radical prostatectomy [18]. Besides, detection of PSA in the sera of PCa patients after radical prostatectomy might be due to the presence of residual benign prostate tissue, which misguides practitioners to come up with a false positive diagnosis [19]. Moreover, patients with similar serum PSA level, Gleason score and pathological stage have been shown to have distinct clinical outcomes due to the heterogeneity of the subtypes at the molecular level [20], [21].

Due to the fact that novel prognostic biomarkers are urgently needed to develop more effective, optimized and individualized therapy strategies, several putative prognostic biomarkers were suggested in recent years, which achieved limited success in patient stratification [22]. For example, various genes, which are specifically detected in the prostate gland, such as human KLK2, PCA3, prostate-specific membrane antigen and prostate stem cell antigen were suggested as useful prognostic markers for prediction of pathological features in PCa patients [23]. Altered expressions of Bcl-2 and Bax were also associated with subsequent development of biochemical recurrence [18]. However, among the genes proposed as prognostic biomarker depending on large-scale gene expression profiling studies, only a few genes could be validated in multiple studies [24]. Moreover, several clinical risk prediction models were developed to predict the biochemical recurrence risk or clinical failure, although these models also failed to reliably and accurately estimate the clinical outcome due to heterogeneity of the disease [25]. Considering the limitations of the current prognostic tools and models, it is important to incorporate novel biomarkers to existing models to overcome these limitations during clinical decision-making processes.

Therefore, it is a vital goal in current prostate cancer research to find out effective prognostic molecular biomarkers that would help accurately identifying patients with aggressive and metastatic disease in order to guide therapeutic decisions and determine the patients who needs closer follow-up and intensive care. In addition, identification of biomarkers having potential to predict recurrence after radical prostatectomy would be of paramount clinical significance to decide whether adjuvant therapy is required. Such biomarkers would be especially invaluable for patients with distinct outcomes although they have similar clinical characteristics.

The first implication of miRNAs in cancer biology has been described through detection of miR-15 and miR-16 downregulation in B cell chronic lymphocytic leukemias [26]. Since then, various miRNAs were associated with tumor pathogenesis through playing role in initiation, progression, and metastasis of cancer [27], [28]. In addition to investigating the differential expression of miRNAs in cancer, it is important to explore miRNA profiles of samples to find out an association of miRNA expression with clinical outcome.

miR-1 and miR-133b, encoded from miR-1/133a and miR-206/133b clusters, were denoted as muscle-specific miRNAs [29] and were reported to be frequently downregulated in various tumor types [30]. Besides, their ectopic overexpression has been demonstrated to inhibit cell growth, cell migration and induce apoptosis in several types of cancers [30]. miR-1 has been recently suggested as a prognostic marker in PCa to predict recurrence [31]. Its downregulation might be involved in PCa recurrence thorough elevated levels of its targets. For example, overexpression of CXCR4 and SDF-1alpha, as validated targets of miR-1 [32], were associated with local recurrence and distant metastasis in PCa [33] and poor prognosis in stage II pancreatic ductal adenocarcinoma [34], respectively. Upregulation of NOTCH3, an oncogenic miR-1 target, has been also demonstrated to associate with PCa recurrence [35]. As to the role of miR-133b in recurrence, it has been associated with overall survival and metastasis in colorectal cancer [36] and proposed as a prognostic biomarker for PCa recurrence in a very recent study [37]. Elevated levels of validated miR-133b targets such as CXCR4 [38], FGFR1 [39], FSCN1 [40] has been associated with prognosis of various cancers.

## Conclusions

The current techniques do not necessarily provide enhanced survival rates for PCa patients and almost 30% of men develop clinical recurrence with elevated serum prostate-specific antigen levels. Therefore, it is of paramount importance to unravel the molecular mechanisms underlying PCa progression to develop novel and effective diagnostic/therapeutic tools. Here, we report that miR-1 and mir-133b have been significantly downregulated in recurrent PCa specimens in comparison to non-recurrent PCa samples, which can serve as novel biomarkers for prediction of PCa progression.

## Supporting Information

Table S1 The list of differentially expressed miRNAs in recurrent vs. non-recurrent prostate cancer samples.
(XLS)
